# Standardization of molecular monitoring of CML: results and recommendations from the European treatment and outcome study

**DOI:** 10.1038/s41375-022-01607-z

**Published:** 2022-05-25

**Authors:** Helen E. White, Matthew Salmon, Francesco Albano, Christina Søs Auður Andersen, Stefan Balabanov, Gueorgui Balatzenko, Gisela Barbany, Jean-Michel Cayuela, Nuno Cerveira, Pascale Cochaux, Dolors Colomer, Daniel Coriu, Joana Diamond, Christian Dietz, Stéphanie Dulucq, Marie Engvall, Georg N. Franke, Egle Gineikiene-Valentine, Michal Gniot, María Teresa Gómez-Casares, Enrico Gottardi, Chloe Hayden, Sandrine Hayette, Andreas Hedblom, Anca Ilea, Barbara Izzo, Antonio Jiménez-Velasco, Tomas Jurcek, Veli Kairisto, Stephen E. Langabeer, Thomas Lion, Nora Meggyesi, Semir Mešanović, Luboslav Mihok, Gerlinde Mitterbauer-Hohendanner, Sylvia Moeckel, Nicole Naumann, Olivier Nibourel, Elisabeth Oppliger Leibundgut, Panayiotis Panayiotidis, Helena Podgornik, Christiane Pott, Inmaculada Rapado, Susan J. Rose, Vivien Schäfer, Tasoula Touloumenidou, Christopher Veigaard, Bianca Venniker-Punt, Claudia Venturi, Paolo Vigneri, Ingvild Vorkinn, Elizabeth Wilkinson, Renata Zadro, Magdalena Zawada, Hana Zizkova, Martin C. Müller, Susanne Saussele, Thomas Ernst, Katerina Machova Polakova, Andreas Hochhaus, Nicholas C. P. Cross

**Affiliations:** 1 Faculty of Medicine, University of Southampton, Southampton, UK; 2 Wessex Regional Genetics Laboratory, Salisbury NHS Foundation Trust, Salisbury, UK; 3 Department of Emergency and Organ Transplantation (D.E.T.O.)—Hematology and Stem Cell Transplantation Unit, University of Bari “Aldo Moro”, Bari, Italy; 4 Department of Pathology, Zealand University Hospital, Roskilde, Denmark; 5 Department of Medical Oncology and Hematology, University Hospital Zurich, University of Zurich, Zurich, Switzerland; 6 Laboratory of Medical Genetics National Specialized Hospital for Active Treatment of Hematological Diseases, Sofia, Bulgaria; 7 Clinical Genetics, Karolinska University Hospital, Stockholm, Sweden; 8 Laboratory of Hematology, University Hospital Saint-Louis, Université de Paris, Paris, France; 9 Department of Genetics and Research Centre, Portuguese Oncology Institute of Porto, Porto, Portugal; 10 Department of Molecular Hemato-Oncology, LHUB-ULB Brussels, Belgium; 11 Pathology Department, Hospital Clinic, Institut d’ Investigacions Biomèdiques August Pi i Sunyer (IDIBAPS), CIBERONC, Barcelona, Spain; 12 Fundeni Clinical Institute, Hematology Department, Bucharest, Romania; 13 Hematology Department, Faculty of Medicine, University of Medicine and Pharmacy “Carol Davila”, Bucharest, Romania; 14 Laboratório de Hemato-Oncologia—LHO Instituto Português de Oncologia Francisco Gentil, Lisbon, Portugal; 15 Institute for Hematology and Oncology (IHO GmbH), Mannheim, Germany; 16 University Hospital of Bordeaux, Laboratory of Hematology, Haut Lévêque Hospital, Pessac, France; 17 Department of Immunology, Genetics and Pathology, Uppsala University, Uppsala, Sweden; 18 University of Leipzig Medical Center, Department for Hematology, Cellular Therapies and Hemostaseology, Leipzig, Germany; 19 Vilnius University Hospital Santaros Klinikos, Vilnius, Lithuania; 20 Poznan University of Medical Sciences, Department of Hematology and Bone Marrow Transplantation, Poznan, Poland; 21 Hematology Department, Hospital Universitario de Gran Canaria Doctor Negrín, Las Palmas de Gran Canaria, Las Palmas, Spain; 22 Laboratory of Chemical and Clinical Analysis “Area 3” A.O.U San Luigi Gonzaga-Orbassano, Turin, Italy; 23 SIHMDS Hosted by Imperial College Healthcare NHS Trust at Hammersmith Hospital, London, UK; 24 Hospices Civils de Lyon, Hôpital Lyon Sud, Service d’Hématologie Biologique, Pierre-Bénite, France; 25 Section of Molecular Diagnostics, Clinical Genetics, Region Skåne, Lund, Sweden; 26 Ritus Biotec Laboratory, Codlea-Brasov, Romania; 27 Transilvania University, Brasov, Romania; 28 Department of Molecular Medicine and Medical Biotechnology University ‘Federico II’ and CEINGE—Advanced Biotechnologies, Naples, Italy; 29 Hematology Department, Hospital Regional Universitario de Málaga, IBIMA, Málaga, Spain; 30 Department of Internal Medicine—Hematology and Oncology, University Hospital Brno, Brno, Czech Republic; 31 Faculty of Medicine, Masaryk University, Brno, Czech Republic; 32 Department of Genomics, Turku University Hospital Laboratories, Turku, Finland; 33 Cancer Molecular Diagnostics, St. James’s Hospital, Dublin, Ireland; 34 Labdia Labordiagnostik/St. Anna Children´s Cancer Research Institute (CCRI), Vienna, Austria; 35 Laboratory of Molecular Genetics, Central Hospital of Southern Pest National Institute of Hematology and Infectious Diseases, Budapest, Hungary; 36 Pathology Department, University Clinical Center Tuzla, Policlinic for Laboratory Diagnostics, Tuzla, Bosnia and Herzegovina; 37 Department of Medical Genetics, National Cancer Institute, Bratislava, Slovakia; 38 Medical University of Vienna, Department of Laboratory Medicine, Vienna, Austria; 39 MLL Munich Leukemia Laboratory, Munich, Germany; 40 III. Medizinische Klinik, Universitätsmedizin Mannheim, Mannheim, Germany; 41 CHU Lille, Laboratoire d’hématologie, F-59000 Lille, France; 42 University Hospital Bern, Bern, Switzerland; 43 University of Bern, Bern, Switzerland; 44 Haematology Research Laboratory, National and Kapodistrian University of Athens, School of Medicine, Athens, Greece; 45 Department of Haematology, University Medical Centre Ljubljana, Ljubljana, Slovenia; 46 Faculty of Pharmacy, University of Ljubljana, Ljubljana, Slovenia; 47 Second Medical Department, University Hospital Schleswig-Holstein, Campus Kiel, Germany; 48 Hematology Department, Hospital Universitario 12 de Octubre, Instituto de Investigación Sanitaria Imas12, 28041 Madrid, Spain; 49 Hematological Malignancies Clinical Research Unit, CNIO, 28029 Madrid, Spain; 50 Centro de Investigación Biomédica en Red de Cáncer (CIBERONC), Instituto Carlos III, 28029 Madrid, Spain; 51 West Midlands Regional Genetics Laboratory, Birmingham Women’s and Children’s NHS Foundation Trust, Birmingham, UK; 52 Abteilung Hämatologie/Onkologie, Klinik für Innere Medizin II, Universitätsklinikum Jena, Jena, Germany; 53 Molecular Diagnostics Laboratory, Hematology Department and HCT Unit, George Papanicolaou General Hospital, Thessaloniki, Greece; 54 HemoDiagnostic Laboratory, Department of Hematology, Aarhus University Hospital, Aarhus, Denmark; 55 Amsterdam University Medical Center, Amsterdam, The Netherlands; 56 IRCSS Azienda Ospedaliero-Universitaria di Bologna Istituto di Ematologia “Seràgnoli”, Bologna, Italy; 57 Department of Clinical and Experimental Medicine, Center of Experimental Oncology and Hematology, University of Catania, Catania, Italy; 58 Molecular Hemapathology, Oslo University Hospital, Oslo, Norway; 59 Haematological Malignancy Diagnostic Service, Leeds Teaching Hospitals, Leeds, UK; 60 University Hospital Center Zagreb, Zagreb, Croatia; 61 The University Hospital in Krakow, Krakow, Poland; 62 Institute of Hematology and Blood Transfusion, Prague, Czech Republic

**Keywords:** Oncogenesis, Prognosis

## Abstract

Standardized monitoring of *BCR::ABL1* mRNA levels is essential for the management of chronic myeloid leukemia (CML) patients. From 2016 to 2021 the European Treatment and Outcome Study for CML (EUTOS) explored the use of secondary, lyophilized cell-based *BCR::ABL1* reference panels traceable to the World Health Organization primary reference material to standardize and validate local laboratory tests. Panels were used to assign and validate conversion factors (CFs) to the International Scale and assess the ability of laboratories to assess deep molecular response (DMR). The study also explored aspects of internal quality control. The percentage of EUTOS reference laboratories (*n* = 50) with CFs validated as optimal or satisfactory increased from 67.5% to 97.6% and 36.4% to 91.7% for *ABL1* and *GUSB*, respectively, during the study period and 98% of laboratories were able to detect MR^4.5^ in most samples. Laboratories with unvalidated CFs had a higher coefficient of variation for BCR::ABL1^IS^ and some laboratories had a limit of blank greater than zero which could affect the accurate reporting of DMR. Our study indicates that secondary reference panels can be used effectively to obtain and validate CFs in a manner equivalent to sample exchange and can also be used to monitor additional aspects of quality assurance.

## Introduction

Molecular monitoring of chronic myeloid leukemia (CML) patients undergoing tyrosine kinase inhibitor (TKI) therapy provides important prognostic information for individual patients and is used to assess time-dependent treatment milestones, including early molecular response (EMR), major molecular response (MMR), and deep molecular response (DMR) [1, 2]. Molecular monitoring is usually performed using reverse transcriptase quantitative PCR (RT-qPCR), which estimates the number of copies of *BCR::ABL1* mRNA relative to those of an internal reference gene, most commonly *ABL1*, *GUSB*, or *BCR*, thus controlling for variation in sample quality and quantity [3, 4]. Current guidelines specify that assay results should be expressed on the International Scale (IS) for *BCR::ABL1* measurement, which is effectively the same as that used in the International Randomized Study of Interferon and STI571 (IRIS). On this scale, 100% BCR::ABL1^IS^ corresponds to the IRIS standardized baseline derived from analysis of 30 pre-treatment chronic phase CML cases. [5] EMR is defined as ≤10% BCR::ABL1^IS^, MMR (also known as MR^3^, i.e., a molecular response of ≥3 logs below the standardized baseline) as ≤0.1% BCR::ABL1^IS^, and levels ≤0.01% (MR^4^) as DMR. [3] Testing laboratories derive results on the IS either by using commercially available kits or systems that have been calibrated to the World Health Organization (WHO) International Genetic Reference Panel for quantitation of *BCR::ABL1* mRNA, or by using a laboratory-developed test (LDT) in conjunction with a laboratory-specific conversion factor (CF) to the IS derived by sample exchange [4, 6–12].

Sample exchange typically involves testing around 30 CML patient samples spanning the range from MR^2^-MR^4.5^ (i.e., 2–4.5 logs below the IRIS standardized baseline) in both an established reference laboratory and a test laboratory followed by calculation of the mean difference by Bland–Altman analysis. The CF is then defined as the multiplication factor needed to correct for the difference [13]. This process has enabled many laboratories with validated CFs to establish themselves as national or regional reference laboratories and then repeat the process of sample exchange, thus propagating CFs to local centers [14]. Although this has worked well for laboratories with tests that are stable over time, it is evident that the establishment and validation of CFs by sample exchange is time consuming, complex, expensive, and can be difficult for smaller laboratories to access [7, 15].

In 2010, the first International Genetic Reference Panel for quantitation of *BCR::ABL1* mRNA was developed as a primary, WHO-accredited standard for IS calibration [8]. The panel is made of lyophilized K562 and HL60 cell line mixtures and therefore incorporates cellular RNA extraction into the IS calibration process. The panel includes four BCR::ABL1^IS^ levels, with different values assigned to each depending on whether *ABL1*, *BCR*, or *GUSB* is used as a reference genes. To conserve this limited resource, the WHO panel is only available to manufacturers of *BCR::ABL1* test kits and secondary standards [15]. In 2016, the first cell-based *BCR::ABL1* secondary reference panel was produced. This is traceable to the WHO panel and has been produced using a similar format (lyophilized K562 and HL60 cell mixes) with the addition of a fifth sample corresponding to MR^4.5^. BCR::ABL1^IS^ values were assigned to the secondary panel using reverse-transcription droplet digital PCR (RT-ddPCR) with reference to *ABL1*, *BCR*, and *GUSB* and the panel was successfully evaluated by 44 different *BCR::ABL1* laboratories [12]. Recently this panel has been commercialized and is now available for laboratories using *ABL1* as a reference gene (AcroMetrix^™^ BCR-ABL Panel, Thermo Fisher Scientific).

In addition to accurate measurement of detectable residual disease, it is also important to ensure that assays are sensitive enough to detect DMR on a routine basis. Many CML patients achieve sustained (>2 years) DMR on TKI therapy and around half remain in treatment-free remission (TFR) after stopping therapy [2, 16]. Standardization of molecular monitoring at deep levels of response is particularly important, not only to meet the recommended criteria for attempting TFR, but also to identify patients showing signs of molecular relapse, for whom DMR is usually regained after rapid resumption of treatment [17].

To maintain confidence in a CF, ensure that *BCR::ABL1* and reference gene assays are stable over time, and monitor the ability of assays to detect DMR, testing laboratories need to perform rigorous internal quality control (IQC) and validate their CF regularly. IQC is important to monitor variation in assay performance over time and ensure that low level *BCR::ABL1* detection is achieved consistently [18]. Branford et al. have recommended the analysis of high (*c*. 10% BCR::ABL1^IS^) and low (*c*. 0.1% BCR::ABL1^IS^) standards on a regular basis, and ideally on every run to check that *BCR::ABL1* and reference gene assays are stable over time [19, 20].

Given the increased technical sensitivity required for low level *BCR::ABL1* detection, a better understanding of the limits of *BCR::ABL1* assay performance is crucial [21]. The limit of detection (LoD) and limit of quantification (LoQ) of a qPCR test is dependent on the background signal (the limit of blank; LoB), which ideally should be zero. Current *BCR::ABL1* RT-qPCR molecular response (MR) guidelines assume that all laboratories are able to detect *BCR::ABL1* with maximal efficiency [17], but this has never been formally tested and it is possible that differences in LoB and LoD for *BCR::ABL1* assays between laboratories result in variation in the way that MR is reported [22].

From 2016 to 2021 the European Treatment and Outcomes Study (EUTOS) for CML has explored the use of the newly available cell-based secondary *BCR::ABL1* reference panels to assign and validate CFs for testing laboratories. In addition, the ability of laboratories to detect MR^4.5^ reliably was assessed and approaches to IQC were explored. Here, we present the results of this study and EUTOS recommendations for ongoing standardization of molecular monitoring for CML using RT-qPCR.

## Methods

### Ability of laboratories to reliably detect MR^4.5^

From 2016–2021 three batches of nine samples were distributed annually (5 distributions) from the Wessex Regional Genetics Laboratory, Salisbury to EUTOS reference laboratories who agreed to participate (2016, *n* = 49; 2017, *n* = 48; 2019, *n* = 50; 2020, *n* = 49; 2021, *n* = 49). Three samples consisted of locally-prepared HL60/K562 cell line mixtures (5 × 10^5^ cells/vial) at approximately 10%, 0.1% and 0.0032% (DMR cell line lysate) BCR::ABL1^IS^ lysed in either Trizol (Thermo Fisher Scientific, Waltham, Massachusetts, USA), RLT (QIAGEN, Hilden, Germany) or Promega Homogenization Solution containing 1-Thioglycerol (Promega, Madison, Wisconsin, USA) according to the preferred RNA extraction method of each center. Plasmid DNA samples (ERMAD623 BCR-ABL pDNA calibrant, Sigma, St. Louis, Missouri, USA) were supplied as a mock “cDNA sample”. Each plasmid sample contained identical and precisely defined *ABL1, GUSB*, and *BCR::ABL1* copy numbers [11] and were used to establish whether *ABL1, GUSB,* and *BCR::ABL1* RT-qPCR assays were performing with equal efficiency. Plasmid samples with different copy numbers were provided for each annual round of testing. Secondary cell-based reference material panels were provided and were composed of five vials of lyophilized cells (HL60/K562) spanning the range 10% - 0.0032% BCR::ABL1^IS^ and supplied by Novartis Pharmaceuticals Corporation (2016–2019) [12] or Thermo Fisher Scientific (AcroMetrix^™^ BCR-ABL Panel, 2020, 2021). Both secondary panels have BCR::ABL1^IS^ values assigned for the reference gene *ABL1* and the Novartis panel also had BCR::ABL1^IS^ values assigned for the reference gene *GUSB*. To enable the AcroMetrix^™^ BCR-ABL Panel to be used to assign CFs to laboratories using *GUSB* as a reference gene, BCR::ABL1^IS^ values were assigned to the batch by calibrating the reagents with the WHO panel at the laboratory in Wessex [8].

All samples were tested using RT-qPCR using standard laboratory protocols following the process shown in Supplementary Fig. 1. To monitor the quality of local routine samples, anonymized reference gene transcript copy numbers were collected for 50 local samples analysed at each laboratory over a 4-week audit period.

### Derivation of conversion factors and monitoring stability over time

CFs were determined using laboratory *BCR::ABL1* results from the secondary reference lyophilized cell line panels using the method described at https://www.nibsc.org/documents/ifu/09-138.pdf; (included in the Supplementary Information along with a CF calculation spreadsheet). The stability of laboratory CFs was assigned annually using the following criteria, which were based on the previously described definition of optimal performance (+/− 1.2 fold difference from reference method) [7], and the observed mean standard deviation in the initial international assessment of the freeze dried cell secondary reference panel (0.2 log/1.6 fold) [12].

Optimal (+/− 1.2 fold): Previous panel CF/New panel CF = 0.83–1.2

Satisfactory (+/− 1.6 fold): Previous panel CF/New panel CF = 0.63–1.58

Unvalidated: Previous panel CF/New panel CF < 0.63 or >1.58

The unvalidated category also included new laboratory assays where there was no existing CF for comparison.

### Monitoring of internal quality control and correlation with stability of conversion factors

We aimed to measure variation in assay performance over time for individual laboratories and assess how this correlated with stability of CFs. We prepared high and low internal quality control standards by making mixtures of HL60 and K562 cell lines (see Supplementary Information) which were stored and distributed as lysates in either Trizol (Thermo Fisher Scientific), RLT (QIAGEN), or Homogenization Solution containing 1-Thioglycerol (Promega). These standards had BCR::ABL1^IS^ values of approximately 5% (high level control) and 0.05% (low level control). Participants were asked to use their established protocols to extract RNA from both controls on a monthly basis, prepare two independent cDNA samples and test by RT-qPCR. Each laboratory submitted a minimum of 12 results from both high- and low-level controls over the 6-month period of the study. Data were submitted for reference gene transcript number, *BCR::ABL1* transcript number, %*BCR::ABL1*/reference gene and BCR::ABL1^IS^ for each IQC sample type. Six batches of high- and low-level control samples were distributed to 46 laboratories and 43 data sets were returned from 41 laboratories at the completion of the study (89%).

### Limit of blank for *BCR::ABL1* RT-qPCR assays

We aimed to determine the LoB for *BCR::ABL1* RT-qPCR in a subset of experienced molecular monitoring laboratories (*n* = 12). The LoB is defined as the highest measurement result that is likely to be observed for a negative sample i.e., the likelihood of reporting a false positive *BCR::ABL1* result at a defined probability (*α*). When *α* = 0.95, the likelihood of a true negative sample giving a result greater than zero (false positive result) is 5%. To determine the LoB, the Clinical and Laboratory Standards Institute guidelines recommend the following minimum requirements: test four negative samples, using two reagent lots of qPCR master mix, on one instrument, on three independent days, analysing two replicates per sample, generating 60 blank replicate results per reagent lot [21]. Prior to the study, a pre-study questionnaire was sent to all laboratories to determine sample requirements (lysis type for subsequent RNA extraction and volume). Fresh (<48 h), 4 ml non-leukemic peripheral blood samples (*n* = 360) were processed and pooled to generate *BCR::ABL1* negative lysates with sufficient *ABL1* copies (Trizol *n* = 4, RLT *n* = 4). *BCR::ABL1* negative samples (*n* = 4) were provided to each participating laboratory. After local RNA extraction and cDNA synthesis, 18 RT-qPCR replicates (15x *BCR::ABL1*, 3x *ABL1*) were performed per sample, per reagent lot using their local standard protocols. Four *BCR::ABL1* negative samples were analysed using two reagent lots of RT-qPCR master mix, on one instrument, on three independent days, analysing two replicates per sample. This generated 144 individual RT-qPCRs in total; 60 *BCR::ABL1* and 12 *ABL1* replicates for each reagent lot (Supplementary Fig. 2). To calculate the LoB for each reagent lot, the *BCR::ABL1* copy number measurements of all samples were ranked in order from lowest to highest X(1), X(2), …, X(60). The rank position corresponding to the chosen value of α was calculated using the equation: “Rank position = 0.5 + (B x *α*)” where B is the number of replicates and α was 0.95. For most laboratories, the rank position was assigned as 57.5 (B = 60). The LoB was the highest measurement value of the sample at the given rank position across both lots.

## Results

### Ability of laboratories to detect MR^4.5^

**Fig. 1:**
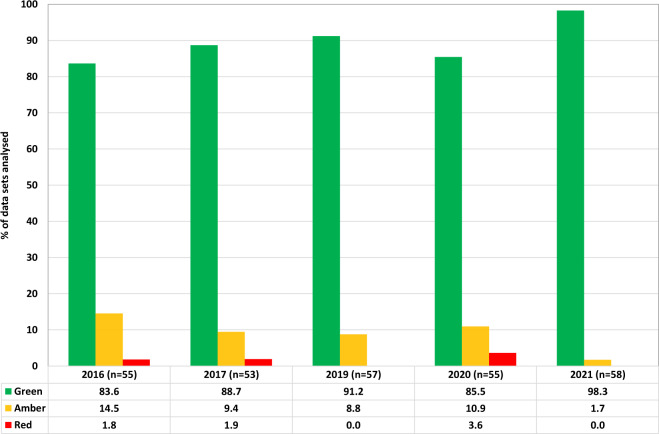
Ability of laboratories to detect MR^4.5^. Overall laboratory scores per reference gene were defined as green (detects MR^4.5^ in a high proportion of samples, combined score > 80%), amber (detects MR^4.5^ in most samples, combined score > 60%) or red (unable to detect MR^4.5^ in most samples, combined score < 60%). The bar charts show the number of data sets in each category for all laboratories. Several laboratories submitted data for more than one reference gene or assay and therefore the number of data sets analysed is greater than the number of participating laboratories.

Analysis of information collected from participating EUTOS reference laboratories showed that there is substantial variation in the methodology used to perform molecular monitoring for CML. Laboratories used different RNA extraction methods, reference genes, PCR machines and RT-qPCR methods (Supplementary Table 1). To assess whether individual laboratories could reliably detect MR^4.5^, data from all test samples were analysed according to five categories of relevant technical measures: (i) median number of reference gene transcripts reported for cell line lysates, (ii) detection of *BCR::ABL1* in the DMR cell line lysates and MR^4.5^ freeze dried reference panel samples, (iii) reference gene and *BCR::ABL1* transcript numbers per µl cDNA, (iv) %*BCR::ABL1*/reference gene for cDNA sample and (v) quality of reference gene audit data. Each category was scored and arbitrarily weighted according to the perceived relevance of each component: cell line results and MR^4.5^ detection> reference gene copy number audit data >cDNA transcript values and cDNA ratio (see Supplementary Table 2 for more details). Combined scores were calculated and an overall laboratory score per reference gene was defined as green (detects MR^4.5^ in a high proportion of samples, combined score >80%), amber (detects MR^4.5^ in most samples, combined score >60%) or red (unable to detect MR^4.5^ in most samples, combined score <60%) as detailed in Supplementary Table 2. The number of data sets in each category, per year, per reference gene are shown in Fig. 1. Several laboratories submitted data for more than one reference gene or assay and therefore the number of data sets analysed is greater than the number of participating laboratories. The categories for cDNA transcript values and cDNA ratios were not scored during the 2021 round due to technical issues. Due to the small sample size and variability of assay conditions it was not possible to observe any significant differences in performance between platform or lysate type.

### Provision of conversion factors and monitoring stability over time

**Fig. 2:**
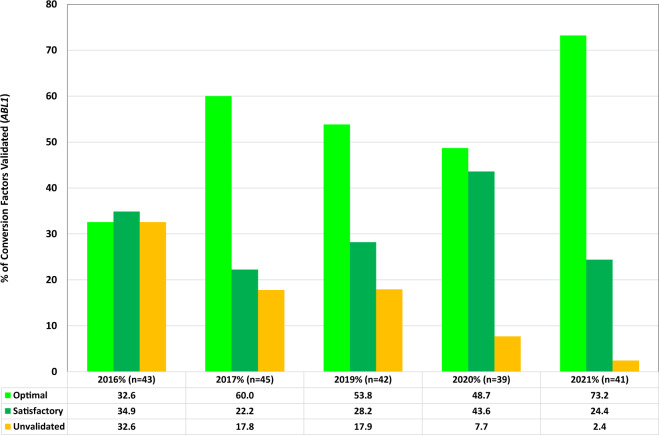
Stability of CFs for laboratories using *ABL1* as a reference gene. CFs were calculated and provided to laboratories on an annual basis. The stability of each CF was determined as either optimal (bright green), satisfactory (green) or unvalidated (amber) by comparison with the previous year’s value using the following criteria; Optimal (+/− 1.2 fold): Old CF/New CF = 0.83–1.2, Satisfactory (+/− 1.6 fold): Old CF/New CF = 0.63–1.58 or Unvalidated: Old CF/New CF < 0.63 or >1.58. The bars charts show the number of laboratories for each category, per year for *ABL1* reference gene data sets. Several laboratories submitted data for more than one assay and therefore the number of data sets analysed may be greater than the number of participating laboratories.

**Fig. 3:**
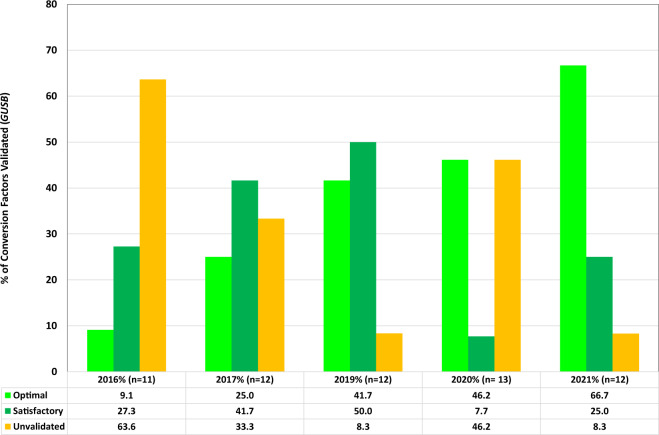
Stability of CFs for laboratories using *GUSB* as a reference gene. CFs were calculated and provided to laboratories on an annual basis. The stability of each CF was determined as either optimal (bright green), satisfactory (green) or unvalidated (amber) by comparison with the previous year’s value using the following criteria; Optimal (+/− 1.2 fold): Old CF/New CF = 0.83–1.2, Satisfactory (+/− 1.6 fold): Old CF/New CF = 0.63–1.58 or Unvalidated: Old CF/New CF < 0.63 or >1.58. The bars charts show the number of laboratories for each category, per year for *GUSB* reference gene data sets. Several laboratories submitted data for more than one reference gene or assay and therefore the number of data sets analysed may be greater than the number of participating laboratories.

CFs were calculated and provided to laboratories on an annual basis for RT-qPCR assays using *ABL1* and *GUSB* as reference genes. The stability of each CF was determined as either optimal, satisfactory or unvalidated by comparison with the previous year’s CF. At the start of the study, laboratories supplied the CF that they were currently using to report BCR::ABL1^IS^ in their laboratory (*n* = 49). Where information was provided (*n* = 41), the laboratory-specific CFs had been obtained using sample exchange from 2014–2016 (93%) or 2012–2013 (7%). Figures 2 and 3 show the number of laboratories for each category, per year, for *ABL1* and *GUSB* reference gene data sets, respectively. Several laboratories submitted data for more than one reference gene or assay and therefore the number of data sets analysed is greater than the number of participating laboratories. The mean, median, maximum, and minimum laboratory CFs for each reference gene per year are shown in Supplementary Table 3. The median CF value from data sets submitted over the course of the study were 0.604 for *ABL1* (interquartile range (IQR) = 0.480–0.780, *n* = 213) and 1.576 for *GUSB* (IQR = 1.16–2.29, *n* = 70) (Supplementary Fig. 3, Supplementary Table 3). This compares to median CFs of 0.563 for *ABL1* (IQR = 0.37–0.81, *n* = 245) and 0.960 for *GUSB* (IQR = 0.68–1.34, *n* = 44) for CFs derived by the EUTOS sample exchange program between 2006 and 2016.

To assess whether the CFs were converting data to the IS reliably, the raw data (%*BCR::ABL1*/reference gene) from each laboratory were converted to the IS using the newly derived CF for the three test samples. For example, in the 2017 round, 72.3% of results were reported within 2-fold of the expected IS value when no conversion factor was applied. This increased to 95.5% of results when data were converted to BCR::ABL1^IS^ using the newly derived CF (Supplementary Table 4). Similar results were seen for all rounds.

### Use of internal quality control material

For the high and low-level standards, the CV was calculated for BCR::ABL1^IS^, total reference gene transcript values and *BCR::ABL1* transcript values for each laboratory. The median, 1st quartile and 3rd quartile CVs for each laboratory and for each parameter are summarized in Table 1.

**Table 1:** 1st quartile, median, and 3rd quartile for the CV (%) values calculated per laboratory for BCR::ABL1^IS^, reference gene copy number, *BCR::ABL1* copy number for the high and low standard.

		High Level IQC Sample CV (%)	Low Level IQC Sample CV (%)
BCR::ABL1^IS^	1st quartile	9.7	14.6
	Median	14.3	21.1
	3rd quartile	22.5	28.9
Reference gene copies	1st quartile	21.8	22.9
	Median	28.2	28.2
	3rd quartile	38.3	35.4
*BCR::ABL1* copies	1st quartile	25.1	26.8
	Median	31.0	33.3
	3rd quartile	38.7	45.6

Overall, the degree of variability for BCR::ABL1^IS^ was comparable to that seen in a previous study [20]. CVs for BCR::ABL1^IS^ determination were used to assess how assay variability might correlate with CF status (optimal, satisfactory, or unvalidated) using data for 2019/2020 since this corresponded to the period when the variability data was collected. The stability of a CF is likely to be affected by variability in assessment of both the high and low standard and therefore we assigned a combined “variability score” using the following criteria:

3 points: CV < 1st quartile

2 points: CV between 1st quartile and median

1 point: CV between median and 3rd quartile

0 points: CV > 3rd quartile

Variability Score (CbVar) = score high level standard + score for low level standard.

**Fig. 4:**
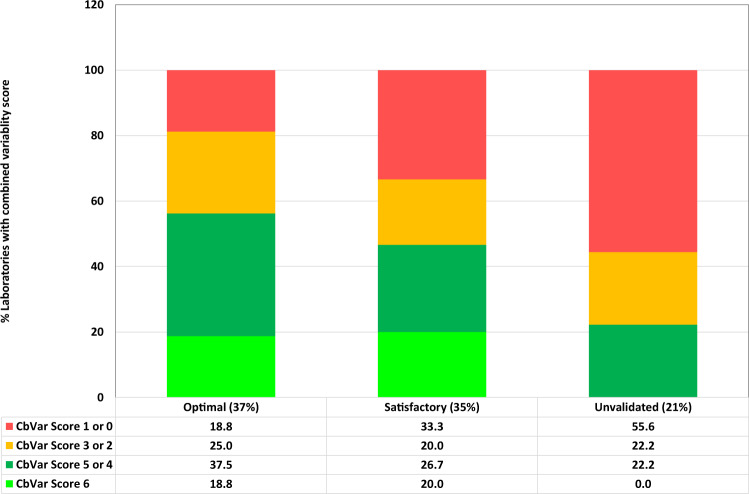
Use of IQC material to assess how CFs correlate with assay variability. CVs for BCR::ABL1^IS^ results from high and low level internal quality control material were used to assess how assay variability might correlate with CF status (optimal, 37% of laboratories who tested the internal quality control material; satisfactory, 35% of laboratories; unvalidated, 21% of laboratories). Combined variability scores for the high and low standards were assigned using the following criteria: 3 points: CV < 1st quartile, 2 points: CV between 1st quartile and median, 1 point: CV between median and 3rd quartile. 0 points: CV > 3rd quartile. The overall variability score (CbVar) was defined as the sum of the scores for the high and low level standards. The bar charts show the % of laboratories per CF status that had a combined variability scores of 6 (bright green). 4 or 5 (green), 2 or 3 (amber) or 1/0 (red).

The data obtained (Fig. 4) shows that 56% of laboratories with unvalidated conversion factors had red variability scores compared to only 19% of optimal laboratories. Overall, there is a clear relationship between variability and CF stability and therefore the BCR::ABL1^IS^ CV of IQC samples is an important quality control metric for laboratories to record routinely.

### Assessment of limit of blank for *BCR::ABL1* detection

For 75% of laboratories (*n* = 9, Laboratories A - I) the likelihood of a true negative sample giving a result greater than zero (i.e., a false positive result) was 5% (Table 2). However, for 25% of laboratories (*n* = 3: laboratories J, K, & L) the likelihood of a true negative sample giving a result greater than zero ranged from 10–50% (Table 2), indicating a significant background of false positive results.

**Table 2:** Limit of blank: data for the 12 participating laboratories.

Lab	A	B	C	D	E	F	G	H	I	J	K	L
Final *BCR::ABL1* LoB (95%)	0	0	0	0	0	0	0	0	0	**0.41**	**2.35**	**2.57**
*Final BCR::ABL1* LoB (90%)	–	–	–	–	–	–	–	–	–	**0**	**0.6**	**2.17**
Final *BCR::ABL1* LoB (85%)	–	–	–	–	–	–	–	–	–	**–**	**0**	**1.95**
Final *BCR::ABL1* LoB (50%)	–	–	–	–	–	–	–	–	–	–	**–**	**0.79**
Total *BCR::ABL1* replicates	120	120	120	120	120	120	90	120	120	**120**	**120**	**120**
No. of negative *BCR::ABL1* replicates	120	120	120	120	120	120	90	119	119	**117**	**110**	**3**
% Negative *BCR::ABL1* replicates	100	100	100	100	100	100	100	99.2	99.2	**97.5**	**91.7**	**2.5**
Max *BCR::ABL1* copy number	0	0	0	0	0	0	0	1.44	2.15	**2.67**	**2.9**	**4.25**

Laboratories A–I have a likelihood of ≤5% that a true *BCR::ABL1* negative sample will give a result greater than zero. Laboratories J, K, and L have a likelihood ranging from 10–50% (indicated in bold) that a true *BCR::ABL1* negative sample will give a result greater than zero.

## Discussion

MRD results directly impact treatment decisions in CML thus it is very important that the accuracy and precision of *BCR::ABL1* assays are maintained across the entire measurement range, and that the sensitivity of the test is sufficient to measure DMR. It is well known that variability exists between RT-qPCR methods [23] and considerable work been undertaken to improve standardization of results for patients with detectable MRD [14, 24], but detailed assessment of the ability of laboratories to detect MR^4.5^ has not been undertaken. Furthermore, the “gold standard” methodology for deriving laboratory-specific CFs by sample exchange has proven to be unsustainable.

The EUTOS molecular standardization study indicates that secondary reference panels can be used effectively to obtain and validate IS CFs over time in a manner equivalent to sample exchange. They can also be used to monitor additional aspects of quality assurance. Over the period of the study, the percentage of laboratories with CFs validated as optimal or satisfactory increased from 67.5% (2016) to 97.6% (2021) and 36.4% (2016) to 91.7% (2021) for *ABL1* and *GUSB*, respectively. The percentage of laboratories able to detect MR^4.5^ in most samples was high across all years with a median of 98.2% (range 96.4 to 100%).

The distribution of *ABL1* CF values was similar to that observed by the EUTOS sample exchange program between 2006 and 2016. However, the distribution of *GUSB* CF values showed an approximate 1.64-fold increase compared to those obtained using sample exchange. It is unclear why this difference occurs, but if the assumption is made that the level of *GUSB* transcripts is 2.4 fold higher than *ABL1*, as shown previously in patient samples [17], then the CF values for *GUSB* assays would be expected to be correspondingly higher. This suggests that the CF values obtained from the cell line panels are valid (*ABL1* median CF 0.604 vs *GUSB* median CF 1.576; 2.6 fold difference). In this study, laboratories using *GUSB* as a reference gene had a higher percentage of unvalidated CFs compared to *ABL1* laboratories (2.4–32.6% *ABL1* vs 8.3–63.6% *GUSB*). The *GUSB* assays also demonstrated a higher degree of variation (mean CbVar = 2) compared to *ABL1* laboratories (mean CbVar = 3.14) when testing internal quality control material, suggesting that the *GUSB* assay may be more inherently variable. It should also be noted that the number of *GUSB* datasets was low for both studies and several *GUSB* laboratories reported technical difficulties using the lyophilized material, possibly due to inexperience in handling this material, resulting in low *GUSB* copy numbers.

Nevertheless, given the potential instability of *GUSB* assays observed in this study, we would suggest that laboratories using this reference gene should monitor the stability of their assays at least monthly using high- and low-level control samples. If instability is detected, the laboratory should consider switching to a validated *ABL1* assay until investigations into the *GUSB* assay stability have been undertaken and successfully actioned. More data are required to fully investigate the use of the panels to derived CFs for *GUSB* assays. Unfortunately, the current AcroMetrix^™^ BCR-ABL Panel has not been calibrated to the primary WHO material for *GUSB* (or *BCR*) and therefore this panel cannot be used to directly derive CFs for this reference gene. Although other approaches may be possible, laboratories using *GUSB* (or *BCR*) as a reference gene will need to continue to perform sample exchange with a reference laboratory to derive a CF. Once a laboratory has established a CF, it should also be possible to revalidate that CF or derive a new CF using archived samples (e.g., lysates) with known IS values that span the range from MR^1^ to MR^4.5^ in a manner analogous to sample exchange with an external reference laboratory.

It is difficult to define exactly how frequently CFs should be revalidated, but we suggest it should be performed at least annually if ongoing IQC data demonstrates assay stability at high and low *BCR::ABL1* values. If the newly derived CF is classified as optimal or satisfactory then it is acceptable to continue to use the original CF, although some centers may prefer to adopt the newly derived CF. However, when a newly derived CF is classified as unvalidated (and the assay has remained unchanged) further investigations should be considered to improve the assay stability. If the method or equipment is changed, or assay drift is noted though ongoing IQC then a new CF will need to be derived [7], although it is important to demonstrate first that any new assay is stable over time. Although we have demonstrated that commercially available secondary reagents can be used to derive a CF, it is important to note that this is not the only option, e.g., sample exchange with a validated laboratory remains an alternative approach, and laboratories may perform their own internal sample exchange, e.g., by comparing results from around 30 stored samples (ideally lysates) spanning 10% to DMR tested with the new method against results from the same samples with the previous, validated method.

For IQC procedures, it is recommended that laboratories attempt initially to optimize assays to decrease variability such that the CV for each category (BCR::ABL1^IS^, reference gene copy number, *BCR::ABL1* copy number) are at least less than the 3rd quartile value obtained in this study (Table 1). Ideally, variability should be close to or lower than the median CV values (Table 1). Once assay variability is established in this range then the application of Westgard rules to accept or reject each run based on the performance of high and low controls (as recommended by Branford et al. [7, 19]) could be used to monitor assays on a regular basis (Supplementary Fig. 4). Laboratories may elect to use a lower standard e.g., 0.01% in addition to, or instead of, 0.1%. The exact level is not critical but we recommend that all laboratories regularly monitor the performance of their assays using at least two standards. Standards may be best prepared locally as lysates of cell line mixtures (see Supplementary Information), or may be purchased from commercial suppliers (e.g., the AcroMetrix^TM^ BCR-ABL panel).

The use of high- and low-level standards can help monitor all the processes in the assay from RNA extraction through to RT-qPCR. Collecting data and monitoring the reference gene number, *BCR::ABL1* copy number and %*BCR::ABL/*reference gene is an ideal way to observe if there are any immediate technical problems occurring with the assay as well as monitoring assay stability over time. Each parameter can provide different information e.g., the copy number information may be useful to determine variability in the cDNA synthesis. In this case, the BCR::ABL1^IS^ may be unaffected but the copy numbers for the reference and target gene may be variable between runs, which may, in turn, affect the LoD. However, if the copy number of one gene is more variable than the other then this may indicate an issue with the RT-qPCR reagents or processes. This would likely affect the BCR::ABL1^IS^ value obtained. For robust IQC, it is therefore recommended to record values for BCR::ABL1^IS^, reference gene copy number, *BCR::ABL1* copy number and also the gradient of the plasmid standard curves and Cq values for each standards on every run. For laboratories using the ERM plasmid the Cq values for *ABL1* and *BCR::ABL1* should be comparable for each standard as the plasmid standard contains exactly the same number of *BCR::ABL1* and *ABL1* copies [11].

Laboratories should be aware of the variability of their assay and communicate this to clinical staff so that they are informed of the acceptable degree of variability of BCR::ABL^IS^ values at critical clinical decision points. For example, a laboratory that has an optimal CV of 9.7% for a high level control sample could reproducibly report a 10% BCR::ABL^IS^ sample in the range of 9.03–10.97% (this range is based on one standard deviation; some laboratories may prefer to use two standard deviations). In the case of a laboratory with an assay demonstrating high variability, e.g., a CV of 22.5%, the range for the same sample increases to 7.75–12.25%. For samples at MMR (0.1% BCR::ABL^IS^) the same laboratories would report a true MMR sample in the range 0.085–0.115% (CV 14.6%) and 0.071–0.129% (CV 28.9%) respectively.

Defining the LoB and LoD of quantitative assays is important for validation of molecular tests and is necessary for accreditation of a diagnostic test to ISO 15189 (2012). Our study provides a practical recommended protocol for determining the LoB for *BCR::ABL1* RT qPCR testing, and we recommend that laboratories establish their LoB. A major challenge was the production of truly *BCR::ABL1* negative samples. Initially, material was prepared from several *BCR::ABL1* negative human cell lines from different sources but in our hands these showed very low level but reproducible amplification with *BCR::ABL1* EAC RT-qPCR assays. Therefore, the use of cell line derived material for LoB studies is not recommended. Preparation of pooled blood samples from non-leukemic patients was time consuming however provided good quality material for the study. Using this material, we found that 25% of laboratories had a LoB greater than zero which may have implications for the accurate reporting of DMR, thus demonstrating the importance of establishing a LoB. Laboratories with poorly optimized assays may either fail to detect *BCR::ABL1* and erroneously conclude that a patient had achieved DMR (variation in LoD) or exhibit a low level false positive rate and erroneously detect *BCR::ABL1* (variation in LoB). Laboratory LoBs and LoDs have not been examined comprehensively to date because of a lack of suitable control reagents and agreed methodology.

In summary, we provide a number of recommendations for optimal monitoring of residual disease in CML by RT-qPCR, including establishment of laboratory-specific CFs and maintenance of reporting on the IS. We anticipate that these recommendations will further help to improve the quality of molecular monitoring for CML, with resulting benefits for patient management.

## Supplementary information

Supplementary Information

Supplementary spreadsheet

## Data Availability

The datasets generated during the study are available from the corresponding author on reasonable request.
