# Supplementary material for: Standardization of molecular monitoring of CML: results and recommendations from the European treatment and outcome study
Source: Leukemia. 2022 May 25;36(7):1834–42. doi: 10.1038/s41375-022-01607-z (PMC9252906; doi:10.1038/s41375-022-01607-z)
Supplement: Supplementary file 1 — Supplementary Information [file 41375_2022_1607_MOESM1_ESM.pdf]

# Standardization of molecular monitoring of CML: results and recommendations from the European Treatment and Outcome Study

## Supplementary information

### Supplementary Methods

**<u>Protocol for preparation of high- and low-level internal quality control cell line samples</u>**

1. Grow enough HL60 and K562 cells for your requirements. We estimate that 5 x $10^5$ HL60 cells per vial give approximately 40,000 *ABL1* copies per assay. A 1:50 dilution of K562 cells will generate a high-level control standard of approximately 10% $BCR::ABL1^{IS}$

2. Spin HL60 cultures at 1500rpm for 15 mins and resuspend pellets in 1 x PBS

3. Count the HL60 cells and resuspend to 1.5 x$10^6$ cells / ml in 1 X PBS

4. Count the K562 cells and spin down required number of cells for a 1:50 dilution.

5. The K562 cells should be resuspended in HL60 cells (1.5 x$10^6$ cells / ml in 1 X PBS) diluting the K562 cells 1:50 to generate the high-level control standard e.g. Spin down 1.5 X $10^7$ K562 cells and resuspend the pellet in 500ml HL60 (7.5 x $10^8$ HL60)

6. The high-level standard can then be diluted 1:100 to generate a low-level control standard of approximately 0.1% $BCR::ABL1^{IS}$. Perform the dilution using the HL60 cells (1.5 x$10^6$ cells / ml in 1 X PBS) as the diluent.

7. For preparation of RLT lysates spin batches of 20ml of culture in 50ml Falcon tubes and resuspend each pellet in 36 ml RLT. The solution should be lysed by shearing with a 20ml syringe and wide gauge needle. This can be aliquoted into 60 vials of 600µl (5 x $10^5$ cells per vial)

8. For preparation of Maxwell (Promega) buffer lysates spin batches of 20ml of culture in 50ml Falcon tubes and resuspend each pellet in of 12ml Maxwell buffer. The solution should be lysed by shearing with a 20ml syringe and wide gauge needle. This can be aliquoted into 60 vials of 200µl (5 x $10^5$ cells per vial)

9. For preparation of Trizol lysates spin batches of 15ml of culture in 50ml Falcon tubes and resuspend each pellet in 45ml Trizol. The solution should be lysed by shearing with a 20ml syringe and wide gauge needle. This can be aliquoted into 45 vials of 1ml (5 x $10^5$ cells per vial).

**Supplementary Table 1:** Details of cell line lysates distributed, control genes analysed, plasmid type used for standard curves, PCR protocol and PCR machines used for all data sets for each year.

| | 2016 | 2017 | 2019 | 2020 | 2021 |
|---|---|---|---|---|---|
| **Lysate** | | | | | |
| RLT | 24 | 23 | 25 | 24 | 29 |
| Trizol | 29 | 28 | 26 | 21 | 19 |
| Maxwell | 0 | 0 | 7 | 11 | 10 |
| **Control gene** | | | | | |
| ABL1 | 41 | 45 | 43 | 42 | 42 |
| GUSB | 11 | 13 | 14 | 14 | 15 |
| GUSB and BCR | 1 | 1 | 1 | 0 | 1 |
| **Plasmid** | | | | | |
| ERM-AD623 | 19 | 21 | 29 | 27 | 28 |
| Ipsogen | 27 | 24 | 25 | 23 | 24 |
| pME-2 | 4 | 4 | 1 | 1 | 1 |
| Other / None | 2 | 2 | 3 | 5 | 5 |
| **PCR protocol** | | | | | |
| EAC | 37 | 35 | 42 | 40 | 38 |
| Ipsogen BCR-ABL1 Mbcr Kit | 7 | 7 | 6 | 6 | 7 |
| Emig | 3 | 3 | 2 | 2 | 2 |
| Other / In house/ Not specified | 6 | 6 | 8 | 8 | 11 |

| | 2016 | 2017 | 2019 | 2020 | 2021 |
|---|---|---|---|---|---|
| **PCR machine** | | | | | |
| ABI StepOne | 2 | 2 | 1 | 1 | 1 |
| ABI 7000 | 1 | 1 | 1 | 0 | 0 |
| ABI 7300 | 1 | 1 | 1 | 1 | 1 |
| ABI 7500 | 12 | 11 | 17 | 16 | 16 |
| ABI 7900 | 5 | 5 | 4 | 2 | 2 |
| BioRad CFX96 | 1 | 1 | 1 | 1 | 1 |
| Bio-Rad QX200 | 1 | 1 | 1 | 0 | 2 |
| LightCycler 1.2 | 3 | 2 | 0 | 0 | 0 |
| LightCycler 1.5 | 1 | 1 | 1 | 0 | 0 |
| LightCycler 2.0 | 3 | 3 | 3 | 3 | 3 |
| LightCycler 480 | 11 | 11 | 9 | 10 | 10 |
| Stratagene MX3005 | 1 | 1 | 1 | 0 | 0 |
| QuantStudio 3 | 0 | 0 | 0 | 1 | 1 |
| QuantStudio 5 | 1 | 3 | 5 | 9 | 9 |
| QuantStudio 7 | 1 | 1 | 3 | 2 | 3 |
| Flex Quant Studio 12k | 0 | 0 | 0 | 1 | 1 |
| RotorGene 3000 | 1 | 1 | 1 | 1 | 2 |
| Rotor Gene 6000 | 6 | 5 | 6 | 5 | 4 |
| Viia 7DX | 2 | 2 | 3 | 2 | 2 |
| Not specified | 0 | 0 | 0 | 1 | 0 |

**Supplementary Table** 2: Scoring criteria for $MR^{4.5}$ detection

| | Category | Score |
|---|---|---|
| 1 | **Median cell line lysate control gene copy number** | |
| | The median total control gene copy number reported was calculated for every replicate of every cell line lysate sample (n=18). A score was assigned based on the deviation from the expected median copy number (ABL1 1.4E+05, GUSB 3.35E+05) | |
| | 2-fold lower than expected value | **6** |
| | 3-fold lower | **5** |
| | 5-fold lower | **2** |
| | >5-fold lower | **0** |
| | | |
| 2 | **% MR4.5 Detection** | |
| | A point was awarded for each MR4.5 sample analysed and detected (n=12, 6 replicates of the 0.0032 $BCR::ABL^{IS}$ cell line lysate sample and 6 replicates of the MR4.5 sample in the secondary reference panel). When a sample failed for technical reasons this was excluded from the analysis. The percentage detection was defined as the number of MR4.5 detected / number of samples analysed | |
| | 100% detected | **6** |
| | >85% detected | **5** |
| | >65% detected | **2** |
| | <65 %detected | **0** |
| | | |
| 3 | **cDNA copy number** | |
| | The mean absolute ABL1 and BCR::ABL1 copy numbers per ul of cDNA analysed were calculated per batch. Each replicate(n=6) was assigned a score based on the deviation from the exact copy number | |
| | 2-fold higher or lower | 3 |
| | 3-fold higher or lower | 2 |
| | 5-fold higher or lower | 1 |
| | >5 fold higher or lower | 0 |
| | The **final cDNA copy number score** was assigned as : 100*(sum of score for all replicates / 18) and scored as follows: | |
| | 100% | **3** |
| | >85% | **2** |
| | >65% | **1** |
| | <65% | **0** |
| | | |
| 4 | **cDNA ratio** | |
| | The % BCR::ABL1/ABL1 for the plasmid sample for each batch was calculated. The plasmid sample contained equal copies of BCR::ABL1 and control genes and therefore the expected % is 100%. Each replicate (n=3) was assigned a score based on the deviation from 100%. | |
| | 80% - 120% | 2 |
| | 60 - 80% or 120% - 140% | 1 |
| | <60% or >140% | 0 |
| | The **final cDNA ratio score** was assigned as : 100*(sum of score for all replicates / 6) and scored as follows: | |
| | 100% | **3** |
| | >85% | **2** |
| | >60% | **1** |
| | <60% | **0** |
| | | |
| 5 | **Audit of control gene values for laboratory samples** | |
| | The percentage of control gene values >32000 (ABL1) or 76,800 (GUSB) were calculated for each laboratory and the following scores assigned: | |
| | >80% of samples | **5** |
| | >60% of samples | **3** |
| | <60% of samples | **0** |
| | | |
| | **FINAL SCORE** | |
| | The final score = 100* (sum of scores for 5 categories / 23) | |
| | **>80%** | **Green: Can detect MR4.5 in a high proportion of samples** |
| | **>60%** | **Orange: Can detect MR4.5 in most samples** |
| | **<60%** | **Red: Unable to detect MR4.5 in most samples** |

**Supplementary Table 3:** Summary statistics for CF values per year from all data sets analysed from 2016 – 2021 for the control genes *ABL1* and *GUSB*

| GUSB CF | 2016 | 2017 | 2019 | 2020 | 2021 | Overall |
|---|---|---|---|---|---|---|
| Mean | 1.704 | 1.885 | 1.698 | 2.076 | 2.042 | 1.891 |
| Median | 1.525 | 1.602 | 1.645 | 1.485 | 1.444 | 1.576 |
| Max | 3.304 | 4.392 | 2.686 | 6.152 | 8.040 | 8.040 |
| Min | 0.914 | 0.585 | 0.904 | 0.906 | 0.965 | 0.585 |

| ABL1 CF | 2016 | 2017 | 2019 | 2020 | 2021 | Overall |
|---|---|---|---|---|---|---|
| Mean | 0.645 | 0.679 | 0.608 | 0.672 | 0.690 | 0.659 |
| Median | 0.648 | 0.600 | 0.547 | 0.623 | 0.647 | 0.604 |
| Max | 1.364 | 1.507 | 1.754 | 1.328 | 1.407 | 1.754 |
| Min | 0.199 | 0.246 | 0.271 | 0.418 | 0.372 | 0.199 |

**Supplementary Table 4:** To assess whether CFs were converting data to the IS reliably, the raw data (%*BCR::ABL1* / reference gene) from each laboratory were converted to BCR::ABL1$^{IS}$ using the newly derived laboratory specific CF. As an example, for the 2017 round 72.3% of results for 3 test samples were within 2 fold of the expected IS value for the raw, unconverted data (left). This increased to 95.5% of results when the data were converted to BCR::ABL1$^{IS}$ using the newly derived CF (right).

| | Raw data %*BCR::ABL1* / *CG* | | | BCR::ABL1$^{IS}$ (2017 CF) | | |
|---|---|---|---|---|---|---|
| **Sample** | **'10%'** | **'MMR'** | **'DMR'** | **'10%'** | **'MMR'** | **'DMR'** |
| IS of Sample | 5.3775 | 0.0534 | 0.0052 | 5.3775 | 0.0534 | 0.0052 |
| 2 fold upper | 10.7550 | 0.1068 | 0.0105 | 10.7550 | 0.1068 | 0.0105 |
| 2 fold lower | 2.6888 | 0.0267 | 0.0026 | 2.6888 | 0.0267 | 0.0026 |
| 5 fold upper | 26.8876 | 0.2669 | 0.0261 | 26.8876 | 0.2669 | 0.0261 |
| 5 fold lower | 1.0755 | 0.0107 | 0.0010 | 1.0755 | 0.0107 | 0.0010 |
| **Lab 1** | 13.6912 | 0.0785 | 0.0054 | 9.7539 | 0.0559 | 0.0038 |
| **Lab 4** | 6.7585 | 0.0517 | 0.0042 | 6.7045 | 0.0513 | 0.0042 |
| **Lab 5** | 3.0189 | 0.0339 | 0.0034 | 4.5502 | 0.0510 | 0.0051 |
| **Lab 6** | 7.2739 | 0.0955 | 0.0114 | 3.8861 | 0.0510 | 0.0061 |
| **Lab 7** | 15.2580 | 0.1526 | 0.0202 | 5.4387 | 0.0544 | 0.0072 |
| **Lab 8** | 9.0541 | 0.0957 | 0.0089 | 5.2426 | 0.0554 | 0.0052 |
| **Lab 9** | 5.9720 | 0.0774 | 0.0084 | 3.7037 | 0.0480 | 0.0052 |
| **Lab 10** | 9.4118 | 0.0953 | 0.0092 | 3.7273 | 0.0377 | 0.0037 |
| **Lab 11** | 12.5683 | 0.1093 | 0.0136 | 4.7884 | 0.0416 | 0.0052 |
| **Lab 12** | 6.3600 | 0.0590 | 0.0061 | 5.5201 | 0.0512 | 0.0053 |
| **Lab 14** | 8.6632 | 0.0833 | 0.0098 | 5.4820 | 0.0527 | 0.0062 |
| **Lab 15** | 13.3761 | 0.1206 | 0.0124 | 5.6868 | 0.0513 | 0.0053 |
| **Lab 16** | 10.7054 | 0.1071 | 0.0111 | 5.8398 | 0.0584 | 0.0061 |
| **Lab 18** | 12.6887 | 0.1360 | 0.0113 | 5.3617 | 0.0575 | 0.0048 |
| **Lab 20** | 8.3192 | 0.1098 | 0.0095 | 4.5667 | 0.0603 | 0.0052 |
| **Lab 21** | 11.9664 | 0.1165 | 0.0096 | 4.9271 | 0.0480 | 0.0040 |
| **Lab 24** | 7.2238 | 0.1345 | 0.0257 | 2.9034 | 0.0541 | 0.0103 |
| **Lab 25** | 6.9125 | 0.0640 | 0.0062 | 5.8869 | 0.0545 | 0.0053 |
| **Lab 26** | 18.3331 | 0.2097 | 0.0224 | 4.5156 | 0.0516 | 0.0055 |
| **Lab 27** | 9.3404 | 0.1160 | 0.0100 | 4.2409 | 0.0527 | 0.0045 |
| **Lab 28** | 4.3193 | 0.0382 | 0.0039 | 5.2528 | 0.0464 | 0.0048 |
| **Lab 29** | 8.7263 | 0.0880 | 0.0101 | 4.7157 | 0.0476 | 0.0055 |
| **Lab 30** | 4.3078 | 0.0383 | 0.0041 | 4.9341 | 0.0439 | 0.0047 |
| **Lab 31** | 8.4226 | 0.0949 | 0.0091 | 3.9898 | 0.0450 | 0.0043 |
| **Lab 33** | 9.9396 | 0.0943 | 0.0122 | 4.6845 | 0.0444 | 0.0057 |
| **Lab 35** | 9.4717 | 0.1017 | 0.0079 | 5.8462 | 0.0628 | 0.0049 |
| **Lab 36** | 6.5303 | 0.0762 | 0.0073 | 4.7161 | 0.0550 | 0.0052 |
| **Lab 38** | 7.6397 | 0.0902 | 0.0091 | 4.3123 | 0.0509 | 0.0051 |
| **Lab 39** | 8.1804 | 0.1197 | 0.0134 | 3.7515 | 0.0549 | 0.0062 |
| **Lab 40** | 8.6677 | 0.0926 | 0.0082 | 6.4750 | 0.0692 | 0.0061 |
| **Lab 41** | 13.2445 | 0.0822 | 0.0047 | 9.2605 | 0.0574 | 0.0033 |
| **Lab 42** | 4.8280 | 0.0373 | 0.0050 | 6.0293 | 0.0466 | 0.0062 |
| **Lab 43** | 7.2066 | 0.0717 | 0.0071 | 4.1715 | 0.0415 | 0.0041 |
| **Lab 45** | 13.6507 | 0.1343 | 0.0152 | 4.9769 | 0.0490 | 0.0055 |
| **Lab 46** | 7.1900 | 0.0598 | 0.0014 | 6.4194 | 0.0534 | 0.0012 |
| **Lab 47** | 10.3390 | 0.1168 | 0.0106 | 4.3562 | 0.0492 | 0.0045 |
| **Lab 49** | 7.4757 | 0.0780 | 0.0075 | 4.2198 | 0.0440 | 0.0042 |
| **Lab 51** | 7.6197 | 0.1094 | ND | 11.0298 | 0.1584 | ND |
| **Lab 52** | 7.2619 | 0.0674 | 0.0062 | 5.3775 | 0.0499 | 0.0046 |
| **Lab 53** | 3.2813 | 0.0539 | 0.0066 | 3.1005 | 0.0510 | 0.0062 |
| **Lab 55** | 6.6773 | 0.0670 | 0.0071 | 6.2374 | 0.0626 | 0.0067 |
| **Lab 57** | 6.1662 | 0.0629 | 0.0061 | 5.0872 | 0.0519 | 0.0051 |
| **Lab 58** | 6.4267 | 0.0692 | 0.0066 | 4.9896 | 0.0537 | 0.0051 |
| **Lab 59** | 6.9851 | 0.0755 | 0.0062 | 4.1912 | 0.0453 | 0.0037 |
| **Lab 60** | 7.4444 | 0.0766 | 0.0053 | 4.9226 | 0.0506 | 0.0035 |
| **Lab 2** | 2.3875 | 0.0236 | 0.0024 | 7.2586 | 0.0718 | 0.0074 |
| **Lab 3** | 4.1636 | 0.0427 | 0.0033 | 7.3370 | 0.0753 | 0.0059 |
| **Lab 13** | 3.7133 | 0.0432 | 0.0041 | 5.9191 | 0.0689 | 0.0066 |
| **Lab 17** | 7.9860 | 0.0550 | 0.0019 | 7.7767 | 0.0535 | 0.0019 |
| **Lab 19** | 2.6474 | 0.0203 | 0.0022 | 11.6270 | 0.0893 | 0.0097 |
| **Lab 22** | 4.3194 | 0.0362 | 0.0030 | 6.7902 | 0.0570 | 0.0047 |
| **Lab 32** | 3.1218 | 0.0377 | 0.0037 | 6.3076 | 0.0762 | 0.0075 |
| **Lab 34** | 5.4980 | 0.0639 | 0.0057 | 5.8364 | 0.0678 | 0.0061 |
| **Lab 37** | 2.6809 | 0.0209 | 0.0020 | 8.3277 | 0.0650 | 0.0062 |
| **Lab 44** | 3.3124 | 0.0309 | 0.0028 | 7.2125 | 0.0673 | 0.0061 |
| **Lab 48** | 4.1096 | 0.0303 | 0.0035 | 6.6150 | 0.0487 | 0.0056 |
| **Lab 50** | 4.7431 | 0.0490 | 0.0043 | 5.4605 | 0.0564 | 0.0049 |
| **Lab 54** | 12.7575 | 0.1849 | 0.0185 | 7.4633 | 0.1081 | 0.0108 |
| **Lab 56** | 4.0030 | 0.0405 | 0.0034 | 5.3932 | 0.0546 | 0.0046 |

**Supplementary Figure 1:** Three batches of samples (n=9) were distributed to each participating laboratory. On Day A, RNA was extracted from 5 vials of the lyophilised cell line secondary reference panel and the 3 cell line lysate samples from Batch 1. The eight RNA samples were divided into two aliquots; one set of samples were stored at -20° and the other were used to synthesize cDNA. RT-qPCR was performed on the eight cDNA samples and the additional 'cDNA' sample from Batch 1 using standard laboratory protocols. On Day B, cDNA was synthesized from the stored RNA from Day A. RT-qPCR was performed on the eight cDNA samples and the 'cDNA' sample from Batch 1 using standard laboratory protocols. After 28 days the whole process was repeated with the samples from Batch 2 (Days C and D) and after a further 28 days the Batch 3 samples were analyzed (Days E and F). The analysis of each batch was separated by 28 days if possible.

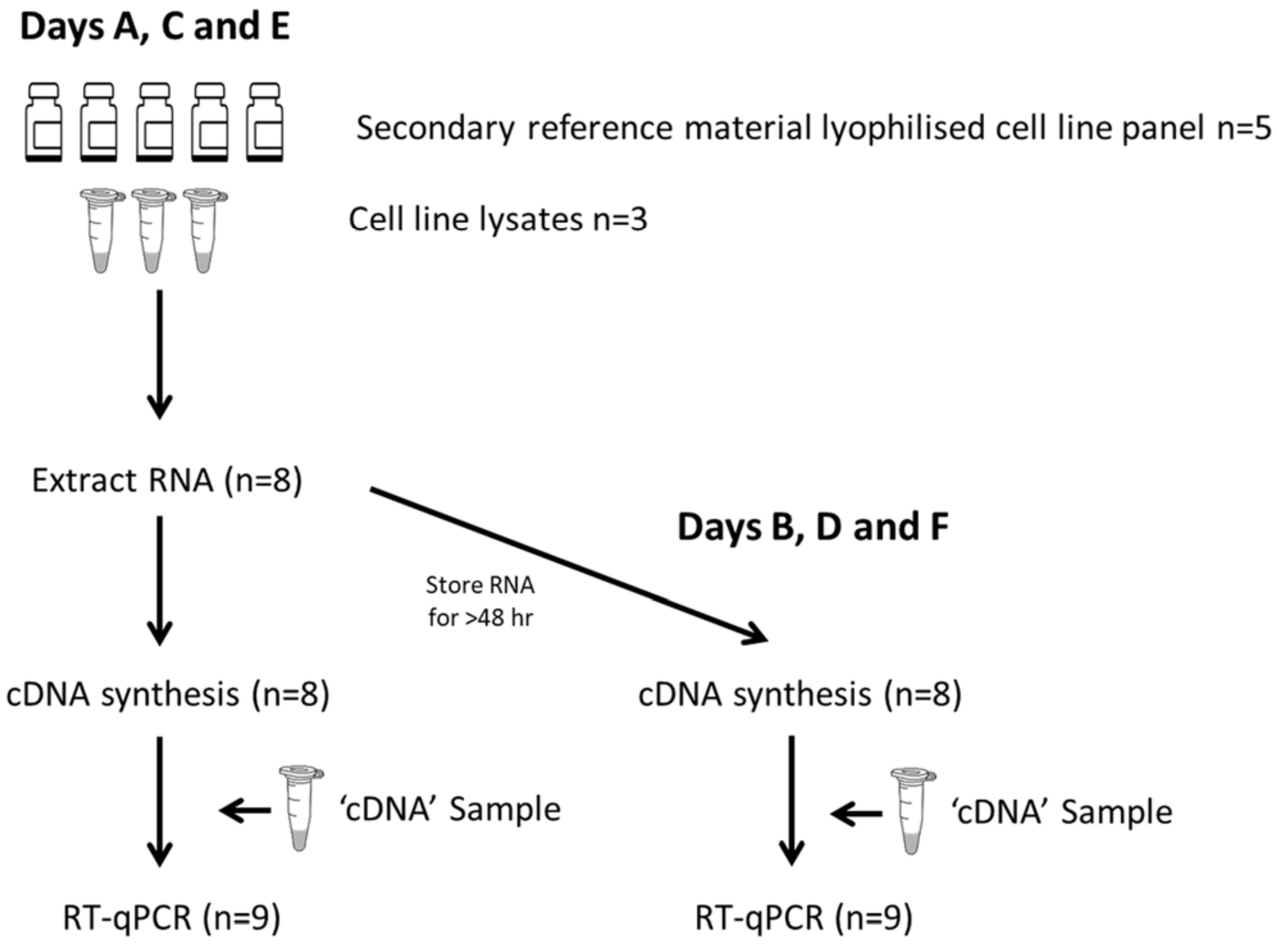

**Supplementary Figure 2:** Four *BCR::ABL1* negative samples were distributed. RNA was extracted from each sample and divided into 2 aliquots. cDNA1 was synthesized from RNA1 on Day A and cDNA2 from RNA2 on Day B. The cell line lysate samples provided sufficient material to generate enough RNA and cDNA to perform 18 RT-qPCR replicates (15 x *BCR::ABL1*, 3 x *ABL1*) per cDNA sample, per reagent lot on three days. (144 individual RT-qPCRs in total; 60 *BCR::ABL1* and 12 *ABL1* replicates for each reagent lot). *ABL1* replicates were included to monitor RNA and cDNA quality.

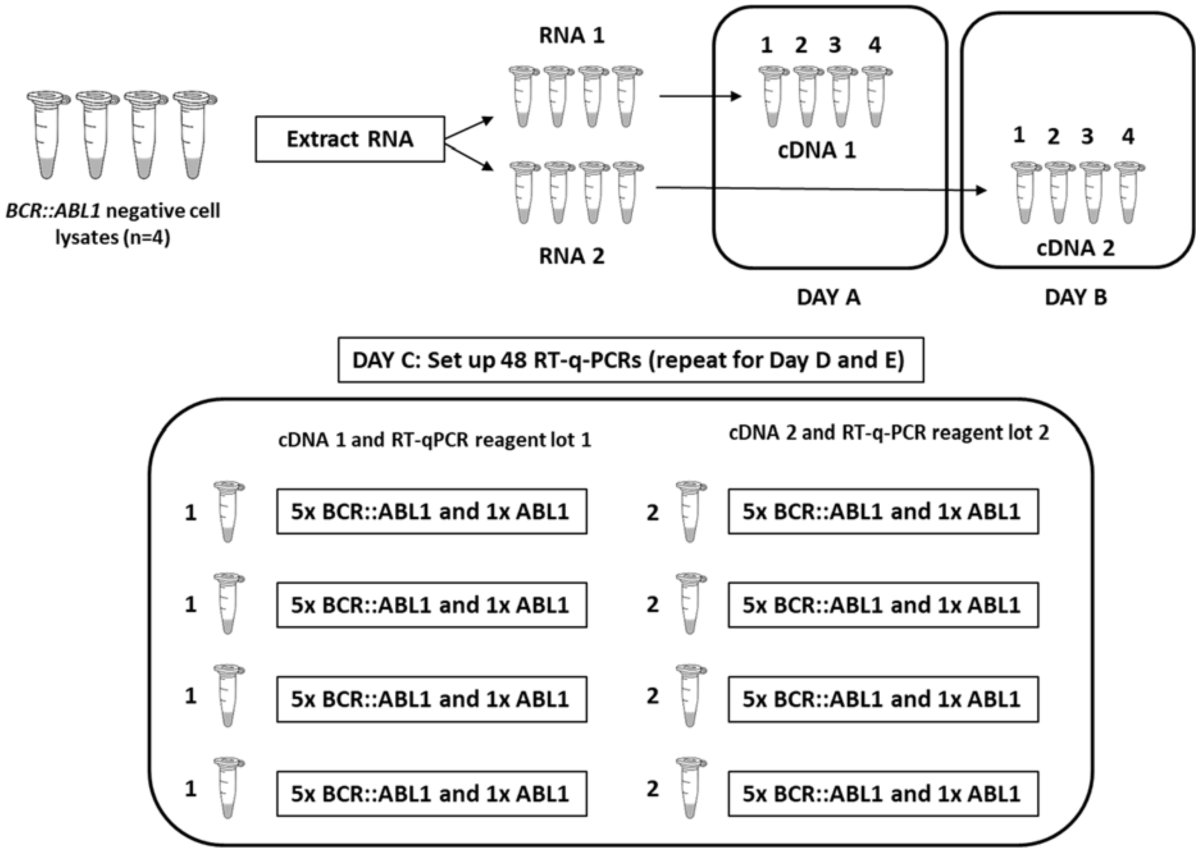

**Supplementary Figure 3:** Box plots and statistics for CF values from all data sets analysed from 2016 – 2021 for the reference genes *ABL1* and *GUSB*

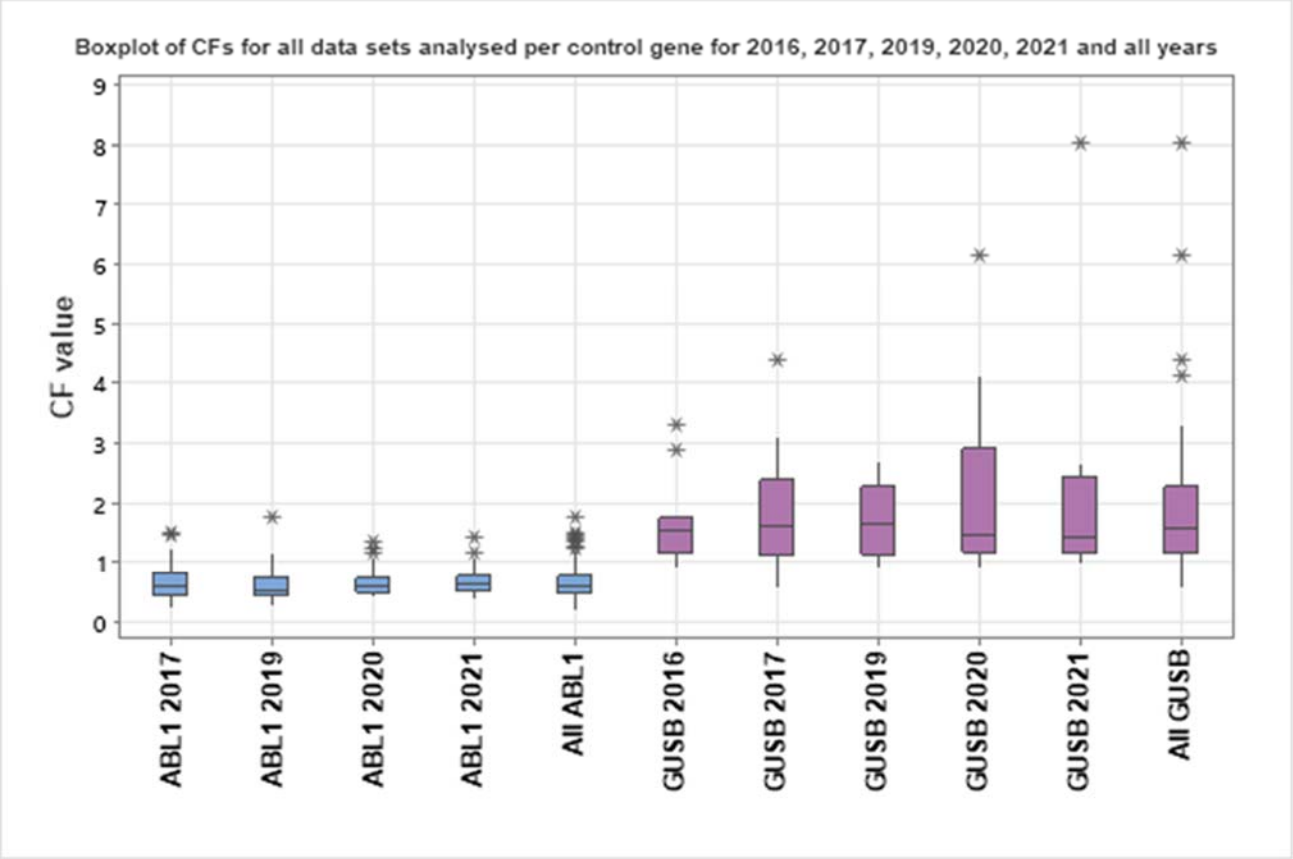

**Supplementary Figure 4.** Application of Westgard rules to accept or reject each run based on the performance of high and low controls (adapted from Branford S, Hughes T. Methods Mol Med 2006;125:69–92; Branford S, et al. Blood 2008;112:3330–3338). Run 2 should be rejected as both the high and low level control results are >2SD from the established mean. Run 9 should be rejected as the high level control result is >3SD.

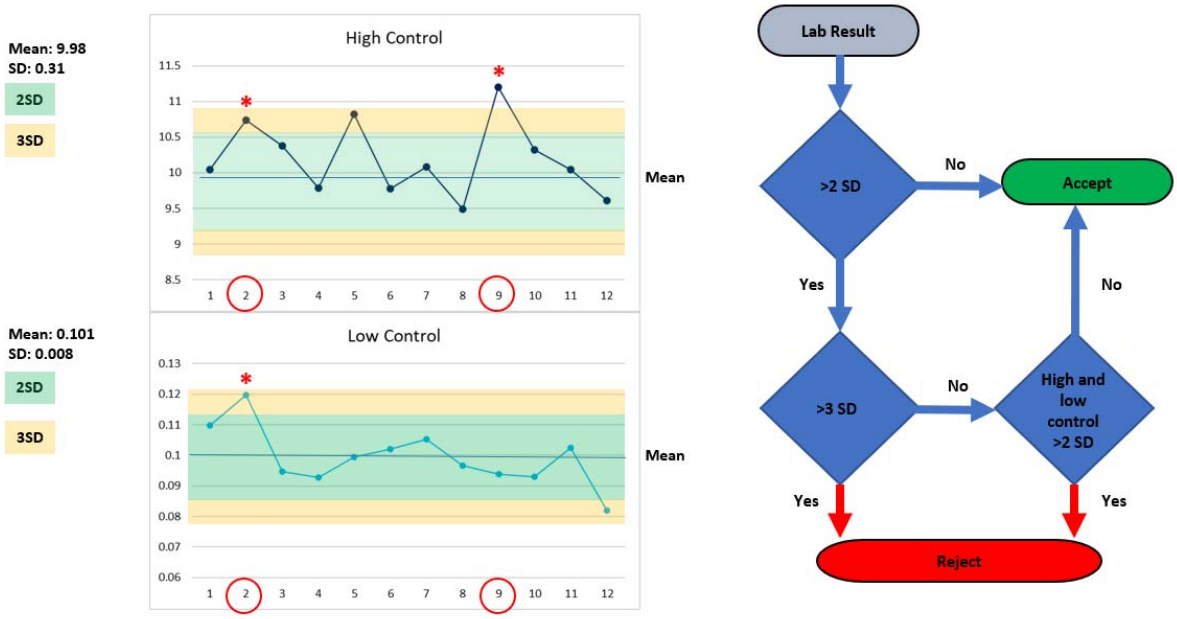

# EUTOS CF Spreadsheet v030322 Instructions for use

The spreadsheet has been produced to help laboratories calculate conversion factors using either sample exchange methods or secondary reference materials. The calculation is based on the linear regression approach suggested by the NIBSC (https://www.nibsc.org/documents/ifu/09-138.pdf; pdf document attached as supplementary material).

**PLEASE NOTE THAT THE SPREADSHEET IS NOT CE MARKED OR OTHERWISE CERTIFIED. IF YOU USE IT TO DERIVE CONVERSION FACTORS FOR CLINICAL USE THEN YOU ARE RESPONSIBLE FOR ENSURING THAT THE RESULTS ARE CORRECT.**

1. Enter the known BCR::ABL1$^{IS}$ value of the reference sample into column A starting in cell A2. The data can be pasted from another spreadsheet

2. Add the corresponding **unconverted** %BCR::ABL1/reference gene value obtained from your analysis of the reference sample into column B starting in cell B2. The data can be pasted in from another spreadsheet

**IMPORTANT: these values should be calculated with NO conversion factor applied.**

3. The spreadsheet allows you to add up to 180 paired values.

**Do not edit or move the data** once they have been added to the sheet. If you need to delete or move data then start a new spreadsheet.

**Do not manipulate the sheet in any way.**

Once all the data are added the **conversion factor** will be displayed in **cell I1**

Three quality parameters are also shown:

1) Lower 95% confidence interval of the slope (I11).

2) Upper 95% confidence interval of the slope (I12).

3) $R^2$ (I14).

Cells I11, I12 and I14 will appear green if the data are linear and show no bias. The 95% confidence interval of the slope must be fully contained within the range 0.83 - 1.20 and the $R^2$ should be >0.97. If the cells appear red then it may be helpful to visualise your data to see where any issues are occurring. For a visual representation of the data the log10 transformed BCR::ABL1$^{IS}$ reference values can be plotted against the corresponding log10 transformed *%BCR::ABL1* / reference gene (laboratory derived non-IS) using the Chart function in Excel.

If you have an existing CF the newly derived CF can be validated using the following criteria:

| | |
|---|---|
| Optimal (+/- 1.2 fold): | Old CF / New CF = 0.83 – 1.2 |
| Satisfactory (+/- 1.6 fold): | Old CF / New CF = 0.63 – 1.58 |
| Unvalidated: | Old CF / New CF <0.63 or >1.58 |
